# Lactic acid bacteria: An annotated selection of World Wide Web sites relevant to the topics in *microbial biotechnology*

**DOI:** 10.1111/1751-7915.12371

**Published:** 2016-06-24

**Authors:** Lawrence P. Wackett

**Affiliations:** ^1^ Department of Biochemistry, Molecular Biology & BiophysicsBioTechnology InstituteUniversity of MinnesotaSt. PaulMN55108USA

## Lactobacillales (lactic acid bacteria):Wikipedia

https://en.wikipedia.org/wiki/Lactobacillales

This page on the lactic acid group of bacteria is heavy on taxonomy.

## Lactic acid bacteria: Textbook of Bacteriology

http://textbookofbacteriology.net/lactics.html

This webpage describes the major taxonomic and metabolic features of lactic acid bacteria.

## Twelve reasons to know about lactic acid bacteria

https://blogs.biomedcentral.com/on-biology/2014/08/29/twelve-reasons-you-need-to-read-about-lactic-acid-bacteria/

Lactic acid bacteria are important in food and also aid in our health and can prevent infections of food by undesirable bacteria.

## Lactic acid bacteria in food

http://www.pickl-it.com/faq/122/what-are-lactic-acid-bacteria/

This article focuses on lactic acid fermentations, that are critical for producing pickles, sauerkraut, cheeses, yogurt, soy sauce, and other foods.

## Lactic acid bacteria:MetaMicrobe

http://www.metamicrobe.com/lactobacteria/

This commercial website offers a reasonable overview of the lactic acid bacteria and their industrial utility.

## Lactic acid bacterial biorefineries

http://www.sciencedirect.com/science/article/pii/S0734975014001104

This review article discusses new generation biotechnological applications for lactic acid bacteria, including the production of polylactides.

## Lactic acid bacteria as modulators of insect health

http://journals.plos.org/plosone/article?id=10.1371/journal.pone.0033188

This study identified bee honey crop and microbiota as a rich source of novel lineages of lactic acid bacteria, notably *Lactobacillus* and *Bifidobacterium* species. These findings have added significance with the current concern over honeybee colony collapse disorder.

## Engineering platform for lactic acid bacteria

http://nar.oxfordjournals.org/content/early/2015/10/25/nar.gkv1093

This paper describes the design and use of tools for modifying lactic acid bacteria. The tools are then used to make variations in the nisin biosynthetic pathway.

## Sensing and consuming lactic acid bacteria

http://www.nature.com/ismej/journal/v10/n3/full/ismej2015134a.html

This interesting article demonstrates that the bacteriovorous nematode worm, *Caenorhabditis elegans*, uses odorant binding proteins to sense lactic acid bacteria by virtue of the diacetal fermentation product that they make as part of their normal metabolism.

## Lactic acid bacteria and skin protection

http://www.medscape.com/viewarticle/828358

Lactic acid bacteria are described here to inhibit a common skin inhabitant, *Staphylococcus epidermidis*.

The latter organism is important to control and it has been shown to be a opportunistic pathogen upon wounding of the skin.

## LAB‐Secretome database

http://www.cmbi.ru.nl/lab_secretome/

This database is stated to serve for comparative analysis of the predicted secretomes of lactic acid bacteria.

## Lactic acid bacterial genomes

http://www.nature.com/ncomms/2015/150929/ncomms9322/abs/ncomms9322.html

This article describes the comparative genomic analysis of 231 lactic acid bacteria.

## Lactic acid bacteria and wine spoilage

http://www.extension.iastate.edu/wine/lactic-acid-bacteria-and-wine-spoilage

Lactic acid bacteria can have various effects on wine, some desirable and some undesirable. They are responsible for malolactate fermentation which can enhance the flavor of wines. In other cases, they can cause extensive wine spoilage.

